# Individual variation in suprasegmental perception: insights from adults with typical hearing and cochlear implants

**DOI:** 10.3389/fpsyg.2025.1652000

**Published:** 2026-01-06

**Authors:** Terrin N. Tamati, Erin M. Ingvalson

**Affiliations:** 1 Department of Speech and Hearing Science, Ohio State University, Columbus, OH, United States; 2 Department of Speech and Hearing Sciences, University of Washington, Seattle, WA, United States

**Keywords:** suprasegmental perception, individual variation, cochlear implants, emotion perception, talker identification

## Abstract

Individuals vary in their ability to perceive suprasegmental cues, such as pitch, intensity, and duration, to make linguistic and nonlinguistic judgments, such as lexical stress, intonation, talker identity, and vocal emotion perception. For adult cochlear implant (CI) users, limitations in pitch perception significantly impair linguistic and nonlinguistic suprasegmental perception, creating barriers to effective real-world communication. While device-related factors are often emphasized in explaining variability in CI outcomes, growing evidence suggests that cognitive-linguistic factors play a critical role in shaping pitch-based suprasegmental perception. In this Perspective, we examine how cognitive-linguistic and experiential factors influence suprasegmental perception in both typically hearing listeners and adult CI users. We argue that these listener-level differences are essential to understanding variability in CI outcomes, offering insight beyond the effects of device limitations. We propose shifting from group-level generalizations to tailored rehabilitation strategies that target individual needs. Potential approaches include segmental speech training, auditory-cognitive training, and targeted pitch perception training. By identifying malleable sources of individual variation, we aim to support more personalized strategies to improve suprasegmental perception for both typically-hearing and hearing-impaired adults.

## 1 Introduction

Suprasegmental features, including pitch, intensity, and temporal components of speech that extend beyond individual sounds and convey critical linguistic and nonlinguistic information. These features allow listeners to distinguish between words, infer emotion, and interpret social context. However, adults with cochlear implants (CIs) often display deficits in suprasegmental perception, posing significant challenges to effective speech communication (Colby and Orena, 2022; Karimi-Boroujeni et al., 2023). Though research has identified group-level challenges, the substantial variability in CI outcomes suggests a need for more personalized approaches.

It is by now well-established that individuals vary in their abilities to learn, perceive, and produce language (Antoniou et al., 2010; Flege et al., 1995; Flege and Fletcher, 1992; MacWhinney, 2009). Research on segmental speech perception, or listeners’ perception of phonemes, has demonstrated individual differences in how listeners process “what is said.” However, the perception of the suprasegmental aspects of speech that convey “how it is said” also varies significantly across individuals (Abu El Adas and Levi, 2022; Adank and Janse, 2010; Bent et al., 2016; Nusbaum and Magnuson, 1997). While prior research on suprasegmental speech perception has largely emphasized talker-level sources of variability, such as prosody, voice characteristics, and accent, less attention has been paid to listener-level factors that shape perception. Among suprasegmental cues, pitch perception is central to both linguistic and nonlinguistic functions and is among the most studied suprasegmental features. In listeners with typical hearing, pitch cues are conveyed through fine spectral detail and temporal periodicity (Oxenham, 2013). In contrast, CIs provide only coarse representations of pitch, largely through temporal cues (Chatterjee and Peng, 2008; Rosen, 1992). These device limitations disrupt access to key suprasegmental information, yet adult CI users show variable outcomes. This individual variability suggests that listener-level cognitive-linguistic and experiential factors interact with degraded input to influence suprasegmental perception in meaningful ways.

In this Perspective paper, we argue that individual differences in the linguistic and nonlinguistic use of pitch are central to advancing theoretical frameworks and clinical approaches for improving communication outcomes in adult CI users. We first review literature on pitch perception by listeners with typical hearing, highlighting how language background, musical training, and domain-general cognitive skills shape suprasegmental perception. We then extend these insights to post-lingually deafened adults who use CIs, exploring how device limitations interact with listener-level factors to shape suprasegmental perception. By linking findings across populations, we aim to identify shared and unique factors contributing to individual variability. These insights will advance our knowledge regarding the mechanisms underlying linguistic and nonlinguistic pitch perception and inform the development of tailored interventions to support communication outcomes.

## 2 Suprasegmental perception among listeners with typical hearing

### 2.1 Linguistic and nonlinguistic pitch perception

Pitch perception plays a critical role in both linguistic and nonlinguistic suprasegmental tasks. For listeners with typical hearing, pitch is derived from both spectral (e.g., harmonic structure) and temporal cues (e.g., periodicity in the temporal fine structure) (Moore, 2008; Oxenham, 2013). These cues support fine frequency resolution as well as the perception of pitch contours that convey suprasegmental information in speech. However, typically-hearing adults vary in how they perceive and use pitch, reflecting differences in auditory and cognitive-linguistic processes.

One source of individual variation among typically-hearing listeners may be response strategies. For example, in a “missing fundamental” task, an artificial stimulus created of frequencies that could be harmonics of some fundamental frequency that is not present in the stimulus (Terhardt, 1979), some listeners perceive the pitch of the stimulus in terms of the missing fundamental, other listeners perceive the pitch in terms of the component frequencies. Ladd et al. (2013) determined this pattern to be a combination of perception and response strategy.

Similarly, in a set of experiments designed to explore individual variation in detecting small pitch changes, Semal and Demany (2006) identified two groups of listeners. The first group had better performance when identifying the direction of pitch change than when detecting that a pitch change occurred. The second group had poorer performance at identification relative to discrimination, and their difficulties with identification occurred when asked to make absolute direction judgments as well as when making same/different judgments. This was also interpreted to reflect response biases, highlighting the interplay between auditory processing and response strategies on pitch perception.

Outside of listeners’ response strategies, musical experience is another factor influencing pitch perception. Musicians consistently outperform non-musicians in pitch-based tasks such as absolute pitch identification, tone language learning, and psychophysical pitch discrimination, likely due to enhanced auditory and cognitive processing (Arndt et al., 2020; Perrachione and Wong, 2007; Van Hedger and Nusbaum, 2018). Cognitive factors such as working memory also contribute to pitch perception. For example, individual differences in auditory working memory capacity have been linked to variability in absolute pitch performance and learning outcomes (Van Hedger et al., 2015). Thus, the musical and cognitive skills the listener brings to the pitch perception task influence performance, with those listeners who have more baseline abilities better able to perceive pitch in more demanding listening situations. Although neither the Ease of Language Understanding model (ELU; Rönnberg et al., 2013) nor the Framework for Understanding Effortful Listening (FUEL; Pichora-Fuller et al., 2016a) specifically address suprasegmental perception, both offer useful perspectives for understanding individual differences pitch-based suprasegmental tasks. The ELU model emphasizes how degraded or ambiguous input may increase reliance on cognitive resources. The FUEL framework proposes how differences in task demands (such as ambiguity in pitch cues) may impose greater listening effort, depending on the listener’s cognitive-linguistic ability and motivation.

Taken together, these findings suggest that pitch perception in typically hearing adults is shaped by the interaction of auditory processing strategies, domain-general cognitive resources, and experiential factors. Importantly, individual differences in pitch perception extend beyond basic frequency discrimination tasks and influence how listeners perceive higher-level suprasegmental features in speech. In the following sections, we examine how pitch contributes to three key domains of suprasegmental perception, specifically, lexical stress, talker identity, and emotion.

### 2.2 Lexical stress perception

Variability in pitch perception plays a critical role in linguistic tasks, such as lexical stress and pitch accent perception. For example, Japanese uses pitch accent wherein the pitch pattern across the morae (syllables) of a word remain consistent regardless of the word’s place in the sentence or the prosodic phrase on the sentence. This is in contrast with English, which uses phrase-level intonation (Pierrehumbert, 1980). English-speaking learners of Japanese show significant variation in pitch accent acquisition (Muradás-Taylor, 2022), with auditory working memory and baseline pitch discrimination predicting learning gains (Goss, 2020). This suggests that the cognitive load associated with pitch perception seems to relate to listeners’ ability to utilize pitch linguistically, at least in the context of learning a new pitch system.

In English, lexical stress differentiates nouns and verbs (e.g., OB-ject vs. ob-JECT), with pitch being one of the most reliable cues, along with intensity and duration (Beckman and Edwards, 1994; Hayes, 1995). Though there is evidence that listeners track and utilize lexical stress to make lexical judgments (Cooper et al., 2002), there has been little investigation into how individual listeners vary in their use of lexical stress cues. Rather, much of the research into individual variation in lexical stress perception has focused on cross-linguistic perception, examining how native and non-native listeners integrate stress and speech segments. In one such study, native English listeners, native Mandarin listeners, and native Korean listeners were tested on the extent to which lexical stress and speech segments together shaped word recognition in English via a visual word paradigm (Connell et al., 2018). Stimuli were selected such that the target and competitor’s first syllables could match both segmentally and suprasegmentally (e.g., carpet and carton), match segmentally but not suprasegmentally (e.g., carpet and cartoon), or match neither segmentally nor suprasegmentally (e.g., parrot and parade). Mismatched segments took advantage of the fact that unstressed syllables have reduced vowels in English. As hypothesized, native English listeners were sensitive to lexical stress and were most advantaged in identifying the target when the suprasegmental and segmental information matched. Native Mandarin listeners, whose language has limited lexical stress, were also sensitive to lexical stress, but only utilized the suprasegmental cue when segmental information was unavailable. Finally, native Korean listeners, whose language does not have lexical stress, showed limited sensitivity to lexical stress, only utilizing the suprasegmental cue when it overlapped with the segmental cue. Similarly, Cantonese listeners outperform English listeners in lexical stress discrimination involving pitch, likely due to their tone language experience, but show no advantage when pitch is absent (Choi et al., 2019).

### 2.3 Talker identity perception

Talker identity perception relies on a combination of suprasegmental cues such as f0, speaking rate, and voice quality, alongside segmental cues. Listeners are sensitive to these talker-specific cues and are able to recognize familiar talkers (Nygaard and Pisoni, 1998; Remez et al., 2007), but individual variability in talker identification further highlights the influence of auditory and cognitive processes.

Abberton and Fourcin (1978) completed one of the earliest studies into talker identification. Using a mix of natural and synthetic tokens, they found that listeners could identify talkers based solely on prosodic information, with fundamental frequency averages and contours emerging as particularly salient cues. However, individual listeners differenced in their cue weighting strategies, with some downweighting fundamental frequency information. Later studies confirmed the reliability of fundamental frequency as a cue to talker identity, showing that listeners could identify talkers even in reversed speech or high- and low-pass filtered conditions (Compton, 1963; Lass et al., 1980; Van Lancker et al., 1985).

Language familiarity also influences talker identification; listeners are more accurate at identifying talkers in their own language than in an unfamiliar language (Goggin et al., 1991; Perrachione and Wong, 2007; Stevenage et al., 2012; Thompson, 1987). Importantly, superior pitch perception appears to be a strong predictor of talker identification ability. Xie and Myers (2015) showed that tone language speakers and musicians – both groups with enhanced pitch perception – performed better in cross-linguistic talker identification tasks relative to English-speaking non-musicians. Thus, it seems that language and musical experience remain the most reliable predictors of listeners’ talker identification performance. Extrapolating from what we learned from variation in pitch perception and lexical stress perception, it is plausible that these experiential factors reduce the cognitive load of the listening task, either directly by enhancing sensitivity to suprasegmental cues or indirectly by facilitating more efficient segmental processing. In turn, reduced cognitive load allows the listener to devote more resources to suprasegmental perception.

### 2.4 Emotion perception

Though the semantic content (meaning conveyed by words and word order) conveys much of a talker’s emotional state, pitch information also plays a key role in emotion perception. Indeed, the emotional prosody and the semantics of the utterance can be in conflict (as in, “I’m fine,” said in a voice indicating the talker is definitely not fine), requiring the listener to utilize both segmental and suprasegmental cues to interpret the talker’s meaning (Bachorowski, 1999; Wurm et al., 2001). Globerson et al. (2013) found that listeners’ pitch perception skills accounted for approximately one-third of the variance in vocal emotion recognition, highlighting the link between pitch perception and emotion perception.

Language background likely influences emotion perception, as seen in lexical stress perception above. Cho and Dewaele found that English and Korean listeners performed similarly when judging congruent emotional prosody and semantics in English sentences. However, when prosody and semantics conflicted, English listeners were better at integrating these cues (e.g., recognizing an utterance such as, “She’s good,” with a negative prosody as potentially being sarcastic), likely due to the greater pitch variation used to convey emotion in English compared to Korean (Cho and Dewaele, 2021). The authors interpreted this as Korean listeners being less able to recognize that the prosody carried semantics that could be distinct from the segmental semantics.

Age also appears to impact vocal emotion perception. Relative to younger adults, older adults have more difficulty identifying spoken emotions, partly due to age-related hearing loss, which reduces access to high-frequency acoustic cues (Amorim et al., 2021; Morgan and Ferguson, 2017). However, age-related declines in vocal emotion perception are not fully explained by age-related hearing or cognitive declines (Dupuis and Pichora-Fuller, 2015; Pichora-Fuller, 2003; Pichora-Fuller et al., 2016a; Ruffman et al., 2009). Older adults show broader difficulties with emotion perception, including interpreting emotional facial expressions (Connolly et al., 2021; Hayes et al., 2020; Vetter et al., 2020). These deficits may reflect a general decline in emotion recognition abilities with age.

Task complexity further exacerbates these challenges. In two experiments designed to explore the effect of sentence context on older adults’ emotion perception, Seddoh et al. (2020) found that older and younger adults were largely equivalent for sentences with a simple subject-verb-object word order. However, older adults were poorer at emotion perception than younger adults for more complex sentences with non-canonical word orders. One interpretation of these findings, then, is that older adults have insufficient cognitive capacity for suprasegmental perception when the processing task is demanding.

### 2.5 Conclusion

Across these studies in pitch perception, lexical stress, talker identity, and emotion perception we see common findings that variability stems from auditory, cognitive and experiential factors. These findings highlight that suprasegmental perception is not simply a function of bottom-up acoustic access, but reflects the listener’s ability to extract and use pitch information in speech. Understanding how these factors drive differences in typically-hearing populations provides a framework for addressing the unique challenges faced by CI users. By identifying shared and distinct factors driving individual outcomes, we can better inform rehabilitation strategies aimed at enhancing suprasegmental perception in both linguistic and nonlinguistic contexts. In the following section, we extend this framework to adult CI users, exploring how listener-level factors shape suprasegmental perception in the context of a degraded auditory signal.

## 3 Suprasegmental perception in adult CI users

Most research on individual differences in speech perception outcomes among adult CI users has focused on segmental speech perception, using isolated word (e.g., CNC words, Peterson and Lehiste, 1962) and sentence recognition tests (e.g., AzBio sentence recognition test, Spahr et al., 2012). In contrast, suprasegmental perception remains relatively understudied, despite its potential to explain the significant individual variability reported in daily listening experiences and communication outcomes among CI users (Boisvert et al., 2020; Lenarz et al., 2017).

Group-level deficits in suprasegemental perception are well established among CI users, with many studies reporting poor or abnormal performance on linguistic and nonlinguistic tasks requiring robust perception of pitch cues. These include lexical tone perception (Fu et al., 2004; Wei et al., 2004), intonation (Marx et al., 2015), talker discrimination and identification (Cullington and Zeng, 2010, 2011; Hay-McCutcheon et al., 2018), voice gender identification (Fu et al., 2004, 2005; Fuller et al., 2014; Massida et al., 2011, 2013; Meister et al., 2016), and vocal emotion perception (Jiam et al., 2017; Luo et al., 2014; Richter and Chatterjee, 2021; Luo et al., 2007), among others. However, performance varies greatly across individuals and tasks, and depends in part on the specific set of cues used to make suprasegemental judgments (Everhardt et al., 2020). Device-level factors, such as electrode array design, insertion depth, channel interaction, and the quality of the electrode-neuron interface, undoubtedly impact access to pitch cues in CI users and help explain these broad group-level limitations (Limb and Roy, 2014). However, these factors alone do not account for the considerable variation observed among individuals with similar devices or audiologic profiles. A complementary approach is to examine how CI users differ in their ability to extract and use degraded pitch cues, drawing on cognitive, linguistic, and experiential resources.

In this section, we review evidence highlighting individual differences in linguistic and nonlinguistic pitch perception among CI users, focusing on how auditory, cognitive, and linguistic factors influence performance. By linking these findings to insights from typically-hearing populations, we aim to identify shared mechanisms and unique challenges that inform more individualized models of communication and rehabilitation in this population.

### 3.1 Linguistic and nonlinguistic pitch perception

Adult CI users experience well-documented challenges in pitch perception, which affects both linguistic and nonlinguistic suprasegmental tasks. Compared to typically-hearing adults, CI users face additional constraints due to the limited fidelity of pitch cues conveyed by the device. In particular, pitch perception in CI users is largely based on temporal envelope cues, which support pitch discrimination up to around 300 Hz. Above this range, temporal cues are less reliable, and the absence of place-based pitch cues further limits access to pitch information (Zeng, 2002). Yet despite these limitations, pitch remains a meaningful source of information, and listeners vary in how they extract and apply this information in everyday communication.

Variability in cue weighting strategies for voice gender perception illustrates the complexity of this issue. At the group level, typically-hearing listeners use both fundamental frequency and vocal tract length (VTL) cues to identify voice gender, whereas adult CI users predominantly rely on fundamental frequency (Fuller et al., 2014; Gaudrain and Başkent, 2018). At the group level, the extent to which CI users rely upon VTL and/or F0 cues may also depend on the type and duration of the stimulus (e.g., word vs. sentence) as well as the social category (e.g., gender vs. age) (Meister et al., 2016; Meister et al., 2020; Schweinberger et al., 2020). Across individual CI users, some CI users make use of VTL cues in addition to fundamental frequency, with variability in cue weighting potentially driven by differences in auditory sensitivity. For example, while many CI users are sufficiently sensitive to fundamental frequency differences to distinguish between typical male and female voices, few demonstrate sufficient resolution to reliably use VTL cues (Fuller et al., 2014; Gaudrain and Başkent, 2018). It remains unclear whether these differences in voice cue sensitivity directly contribute to cue weighting, or whether listeners’ perceptual strategies also reflect adaptation to degraded input. Understanding these strategies could inform training approaches that support flexible cue use and help CI users more effectively leverage available suprasegmental information.

Residual acoustic hearing offers additional insights into variability in pitch perception. Due to the broadening of candidacy criteria, improved surgical techniques, and changes to electrode designs, many adult CI users are able to retain aidable residual low-frequency acoustic hearing, either in non-implanted (“bimodal” configuration) or implanted ear (electric-acoustic [EAS] stimulation). Residual hearing typically enhances performance in pitch-based tasks, including lexical tone, stress, intonation, and talker identity perception (Hay-McCutcheon et al., 2018; Luo et al., 2014; Marx et al., 2015; Most et al., 2011; Zhou et al., 2020). However, this benefit varies widely across studies and individuals, with some studies finding little to no bimodal advantage (Abdeltawwab et al., 2016; Cullington and Zeng, 2010, 2011). While auditory and device-related factors, such as residual hearing thresholds, electrode configuration, and integration of acoustic and electric input, likely influence the extent of benefit from residual hearing (Payne et al., 2023), emerging evidence suggests that cognitive factors may also shape listeners’ ability to make use of acoustic and electric cues during pitch-based tasks (Hua et al., 2017; Nyirjesy et al., 2024). Thus, further research is needed to understand how residual hearing interacts with auditory and cognitive factors to shape listening outcomes.

### 3.2 Talker identity perception

Adult CI users often face challenges in differentiating and identifying talkers relative to their typically-hearing peers (for a review, see Colby and Orena, 2022). While group-level deficits are well established, performance varies considerably across individuals, with some CI users potentially relying more heavily on cognitive resources. For example, Tamati et al. (2024) found that, at the group level, CI users demonstrated greater sensitivity and less bias when processing lexically easy words (high frequency, low neighborhood density) compared to lexically hard words (low frequency, high neighborhood density). At the individual level, performance varied substantially, with some CI users maintaining relatively stable accuracy across lexical difficulty, while others showed pronounced declines for lexically hard words. Exploratory analyses further revealed that bilateral and bimodal CI users demonstrated greater sensitivity in discriminating same-gender talkers, particularly for lexically hard words, compared to unilateral CI users. These findings suggest that access to additional acoustic information may enhance the ability to utilize top-down linguistic cues in suprasegmental tasks.

Other studies have more directly investigated the contribution of cognitive-linguistic abilities to talker perception. Li et al. (2022) reported that talker discrimination accuracy was related to several core cognitive functions, including inhibitory control, speed of lexical access, and nonverbal reasoning. These findings provide further support for the idea that suprasegmental perception is shaped not only by auditory input but also by cognitive-linguistic ability in adult CI users. Further, they align with cognitive hearing sciences frameworks such as ELU and FUEL (Rönnberg et al., 2013; Pichora-Fuller et al., 2016a), which propose that listeners rely more upon cognitive-linguistic resources when listening conditions are suboptimal. While these frameworks have been largely applied to broader speech understanding outcomes, they may also be useful for understanding and explaining individual differences in talker perception, as well as other pitch-based suprasegmental linguistic and nonlinguistic tasks. Taken together, this body of work highlights the importance of considering both auditory and cognitive-linguistic factors when evaluating individual differences in talker perception among adult CI users.

### 3.3 Lexical stress perception

Suprasegmental information, including pitch, intensity, and duration, not only support talker identification, but also serve linguistic functions such as emphasizing certain syllables to distinguish individual words and determine meaning (i.e., lexical stress), emphasize words in sentences to convey meaning (i.e., sentence stress), or to communicate grammatical information of a phrase or sentence (i.e., intonation). In English, stressed syllables are typically marked by higher pitch, greater intensity, longer duration, and fuller vowel quality, while vowels in unstressed syllables are often reduced (Bolinger, 1961; Lehiste, 1970). Typically-hearing listeners rely most heavily on vowel quality for lexical stress perception, followed by pitch, intensity, and duration (Chrabaszcz et al., 2014). In contrast, CI users generally demonstrate weak or abnormal lexical stress perception (Dincer D’Alessandro and Mancini, 2019; Everhardt et al., 2020; Morris et al., 2013; Most et al., 2011).

Recent work has begun to clarify the perceptual strategies used to perceive lexical stress by CI users. Fleming and Winn (2022) found that CI users relied more on vowel duration and intensity cues and less on pitch and vowel quality, compared to typically-hearing adults. Interestingly, CI users used both duration and pitch to a greater extent than the typically hearing adults listening to fully vocoded speech, suggesting that long-term experience with degraded signals may lead CI users to adopt more flexible cue weighting strategies. Simulations of bimodal hearing indicated low-frequency acoustic information could enhance reliance on pitch and vowel quality. Meister et al. (2011) examined sentence stress perception in adult CI users. They found that CI users were less sensitive to pitch and intensity than typically-hearing listeners, but performed similarly on duration-based tasks. Interestingly, discrimination of covarying pitch and intensity changes was more strongly correlated with identification of sentence stress in natural speech, compared to duration or pitch or intensity alone, suggesting that the use of both cues may help to compensate for degraded access to individual prosodic cues. These findings point to individual differences in auditory access and adaptive cue weighting as key contributors to variability in lexical stress and sentence stress perception among CI users.

Adult CI users similarly demonstrate relatively weak or abnormal intonation perception compared to typically-hearing peers. For example, Marx et al. (2015) found that CI users with residual hearing (thresholds better than 60 dB in the lower frequencies, 125 to 500 Hz), who were tested in their best-aided configuration, had better question/statement discrimination than CI users without any functional residual hearing, although both performed worse than typically-hearing adults. Sensitivity declined when pitch was removed, suggesting that pitch plays a role for these groups. Further, for the CI users with residual hearing, mean residual hearing level and performance on an f0 discrimination task were related to question/statement discrimination, reinforcing the critical role of pitch. Yet, it is important to note that reliance on secondary cues may increase susceptibility to additional degradations in challenging listening environments (Peng et al., 2008) and may lead to increased listening effort (Amichetti et al., 2021).

### 3.4 Emotion perception

Emotion perception presents another significant challenge for CI users. Luo et al. (2007) found that whereas listeners with typical hearing identified emotions with near-ceiling accuracy (89.9%) in a five-alternative forced-choice task, CI users, tested with only their implants, achieved much lower but above chance performance (44.9%). Similar findings across studies using different methodological approaches (Chatterjee et al., 2015; Jiam et al., 2017; Richter and Chatterjee, 2021) confirm that deficits in pitch perception substantially impact emotional prosody perception.

To compensate, CI users may rely on suprasegmental cues that are more reliably conveyed by the device, such as intensity and duration cues, or leverage linguistic knowledge. For example, Richter and Chatterjee (2021) showed that CI users relied more heavily on lexico-semantic cues when prosodic and semantic cues to emotion were incongruent, whereas typically-hearing listeners relied heavily on prosodic cues. Similarly, Taitelbaum-Swead et al. (2022) reported that CI users with stronger prosody-based emotion identification also showed stronger intonation perception, highlighting the shared reliance on pitch in both tasks.

Research on the perception of speaker sincerity, closely linked to emotion, offers additional insights into individual differences. Rothermich et al. (2022) showed that while CI users had difficulty identifying insincere speech, such as sarcasm or teasing, their performance improved with visual cues or verbal context. Bimodal users (CI + hearing aid) were more accurate at identifying speaker sincerity in the auditory-only condition, likely due to stronger pitch perception. Importantly, CI users who benefited more from visual cues showed more accurate understanding of the content of the conversation in the auditory-only condition. This suggests that when speech understanding is less effortful, listeners may be better able to allocate cognitive resources toward integrating multimodal cues. These findings further emphasize the importance of considering cognitive-linguistic factors in explaining variability in emotion perception among adult CI users.

### 3.5 Conclusion

This section illustrates how auditory and cognitive-linguistic factors jointly shape suprasegmental perception in adult CI users. Across tasks involving lexical stress, talker identity, and emotion perception, we see that individual outcomes reflect not only the degraded nature of CI input but also variability in listeners’ perceptual strategies and cognitive-linguistic skills. These findings suggest that optimal (re)habilitation strategies may need to be tailored to each listener’s unique combination of hearing configuration, auditory sensitivity, and cognitive strengths. Leveraging top-down strategies, such as linguistic content and visual cues, may help CI users mitigate challenges in pitch-based suprasegmental perception and support more effective communication in real-world settings. In the next section, we will build on these findings to consider how auditory training approaches can be designed to support suprasegmental perception.

## 4 Future rehabilitation targets

Before suggesting possible rehabilitation targets, we wish to emphasize that this is a perspectives article. Though we have made every effort to provide an overview of the literature regarding suprasegmental perception, we did not conduct a meta-analysis of the available data nor evaluate the quality of the literature. As a result, some of the possible rehabilitation targets may prove to be more efficacious than others, and we encourage research to rigorously test the efficacy of approaches to improve suprasegmental perception for listeners who use CIs.

While bottom-up auditory factors, including device-level constraints and listener-specific hearing characteristics, limit access to pitch cues, top-down cognitive factors offer a promising target for improving suprasegmental perception through training. For both the typically-hearing listeners and CI users, an increase in cognitive load may occur when the talker’s language background differs from that of the listener’s or when the speech signal is degraded. There is evidence indicating that listeners are more successful perceiving suprasegmental information when the listening load is lower (Xie and Myers, 2015), and increases in cognitive load are therefore thought to directly contribute to difficulties with suprasegmental speech perception. When listeners devote all their cognitive resources to accurately perceiving the segmental information, there are few resources available to perceive suprasegmental information (Ip and Cutler, 2020; Seddoh et al., 2020). To that end, rehabilitation efforts that can reduce processing demands may indirectly lead to improvements in the perception of suprasegmental information.

If it is correct that CI users are devoting most of their cognitive resources to perceive segments (Ip and Cutler, 2020), automizing the perception of segments should free up resources for suprasegmental pitch perception (Pichora-Fuller et al., 2016b; Rönnberg et al., 2013). Insights from the L2 literature have shown that training can lead to improvements in L2 speech perception accuracy, though outcomes are variable with some listeners showing larger training gains than others (for reviews see Ingvalson et al., 2014; Ingvalson and Wong, 2016). One way to reduce post-training outcome variability is to match training paradigms to listeners’ baseline abilities (Chandrasekaran et al., 2010; Golestani and Zatorre, 2009; Ingvalson et al., 2013a). Applying this approach to speech segment training in CI users, Moberly and colleagues found that phonological awareness training (i.e., rhyme detection, phoneme matching, and sound blending) led to improved speech perception accuracy in CI users with weak phonological sensitivity (Moberly et al., 2017). Future work could apply other lessons learned from the L2 speech training literature to further automize speech segment perception for CI users.

Another approach involves auditory-cognitive training, which combines auditory skills (e.g., gap detection, phoneme recognition) with domain-general cognitive abilities (e.g., working memory capacity). Though brain training in general has not been demonstrated to be effective at improving everyday cognitive performance (Simons et al., 2016), there is evidence that auditory-cognitive training can improve listeners’ speech perception (Ingvalson et al., 2015; Lelo de Larrea-Mancera et al., 2022; Mishra and Boddupally, 2018). These training paradigms are thought to improve processing capacity and efficiency, reducing the cognitive load necessary for speech perception and freeing up cognitive resources for other linguistic tasks, including suprasegmental pitch perception (Ingvalson and Wong, 2013). In CI users, auditory-cognitive training has been shown to improve speech perception in noise (Ingvalson et al., 2013b; Mishra et al., 2015). Such training, potentially improving speech processing efficiency and capacity, may lead to improved suprasegmental perception. Future work in this area could explicitly explore the impacts of auditory-cognitive training on suprasegmental tasks.

Finally, CI users’ pitch perception may be directly trainable, as evidenced by lexical tone training in prelingually deafened Mandarin-speaking children. Interestingly, these efforts have also borrowed from the L2 speech training literature, capitalizing on efforts to teach native English speakers lexical tone (Cooper and Wang, 2013; Wang et al., 1999, 2003). These studies have found that training paradigms that utilize multiple talkers, include lexical tone in multiple phonetic contexts, and provide feedback on children’s responses are effective in improving lexical tone perception (Zhang D. et al., 2023; Zhang H. et al., 2023; Zhang et al., 2021, 2024). While promising, these training paradigms have primarily been developed for typically-hearing L2 learners or pediatric CI users, and their efficacy in adult CI users has not been established. Differences in linguistic development, auditory plasticity, and cognitive abilities across populations highlight the need for adult-specific research. Adapting these methods for adult CI users, future studies could investigate how training programs tailored to baseline abilities impact pitch perception and whether these improvements translate to better suprasegmental processing. One exciting possibility for this avenue of research is that directly targeting and improving pitch perception could increase the automaticity of suprasegmental perception (Leitman et al., 2009), which in turn could free up cognitive resources for segmental perception and meaning comprehension, or simply alleviating listening-related fatigue.

## 5 Conclusion

Understanding variability in suprasegmental perception is critical for advancing both theoretical frameworks and clinical outcomes in CI users. We note that much of the suprasegmental research to-date has focused on *talker* level sources of variation; in this point of view paper we highlighted *listener* level sources of variability as rehabilitation is more likely to take place at the individual listener level. Variability in lexical stress, talker identity, and emotion perception demonstrates the interplay between auditory limitations and individual factors, such as residual hearing, language background, and cognitive abilities. These differences highlight the inadequacy of one-size-fits-all approaches in auditory rehabilitation and emphasize the importance of tailoring interventions to individual needs.

An increased focus on individual differences offers an opportunity to develop more effective rehabilitation strategies. In the final section we highlighted research suggesting segmental speech training, auditory-cognitive training, and/or pitch perception training may be effective for improving pitch-based linguistic or nonlinguistic perception. However, much of this work has been done in either typically-hearing adults learning an L2 or in pediatric CI users, meaning it remains unknown how effective these training paradigms will be for adult CI recipients, who were the focus of this paper.

A shift in our approach to rehabilitation for adult CI users is needed. Moving beyond group-level generalizations to embrace individual profiles will enable more targeted and effective interventions. The rapid advancements in artificial intelligence provide an exciting opportunity to personalize training approaches based on a given CI-user’s strengths and weaknesses. To support such personalized training, we encourage others in the field to help close the gap in research regarding listener-level sources of variability, and their malleability. This knowledge combined with new technologies have the potential improve outcomes for both typically-hearing listeners and hearing-impaired listeners who struggle with pitch-based suprasegmental perception, and we are excited to see what the future holds.

## Data Availability

The original contributions presented in the study are included in the article/supplementary material, further inquiries can be directed to the corresponding author.
